# Interfacial dominated ferromagnetism in nanograined ZnO: a μSR and DFT study

**DOI:** 10.1038/srep08871

**Published:** 2015-03-09

**Authors:** Thomas Tietze, Patrick Audehm, Yu–Chun Chen, Gisela Schütz, Boris B. Straumal, Svetlana G. Protasova, Andrey A. Mazilkin, Petr B. Straumal, Thomas Prokscha, Hubertus Luetkens, Zaher Salman, Andreas Suter, Brigitte Baretzky, Karin Fink, Wolfgang Wenzel, Denis Danilov, Eberhard Goering

**Affiliations:** 1 Max-Planck-Institute for Intelligent Systems, Heisenbergstr. 3, D-70569 Stuttgart, Germany; 2 Moscow Institute of Physics and Technology (State University), Institutskii per. 9, 141700 Dolgoprudny, Russia; 3 Institute of Solid State Physics, Russian Academy of Sciences, Ac. Ossipyan str. 2, 142432 Chernogolovka, Russia; 4 National Research Technological University “MISiS”, Leninsky prosp. 4, 119991 Moscow, Russia; 5 Laboratory for Muon Spin Spectroscopy, Paul Scherrer Institut, CH-5232 Villigen, Switzerland; 6 Karlsruhe Institute of Technology, Institute of Nanotechnology, Hermann-von-Helmholtz-Platz 1, D-76344 Eggenstein-Leopoldshafen, Germany; 7 A.A. Baikov Institute of Metallurgy and Materials Science RAS, 117991 Moscow, Russia

## Abstract

Diamagnetic oxides can, under certain conditions, become ferromagnetic at room temperature and therefore are promising candidates for future material in spintronic devices. Contrary to early predictions, doping ZnO with uniformly distributed magnetic ions is not essential to obtain ferromagnetic samples. Instead, the nanostructure seems to play the key role, as room temperature ferromagnetism was also found in nanograined, undoped ZnO. However, the origin of room temperature ferromagnetism in primarily non–magnetic oxides like ZnO is still unexplained and a controversial subject within the scientific community. Using low energy muon spin relaxation in combination with SQUID and TEM techniques, we demonstrate that the magnetic volume fraction is strongly related to the sample volume fraction occupied by grain boundaries. With molecular dynamics and density functional theory we find ferromagnetic coupled electron states in ZnO grain boundaries. Our results provide evidence and a microscopic model for room temperature ferromagnetism in oxides.

Several theoretical works predicted the possibility of magnetic doping of semiconducting materials^1^. Soon thereafter magnetism in a large variety of differently doped semiconducting host materials was reported^2^. The idea was that a slight (~ 5%) transition metal (TM) doping of a semiconductor like ZnO would lead to ferromagnetic coupling of these ions within the host material, even at room temperature (RT). Magnetization loops from bulk measurement devices, like a superconducting quantum interference device (SQUID), showed ferromagnetic-like magnetization loops with large magnetic moments per doping ion. However, attempts to attribute this magnetism to the TM ions with element specific methods like x-ray magnetic circular dichroism (XMCD) showed either only paramagnetic behavior^3^,^4^,^5^ or cluster formation of the TM ions^6^. Later on, an increasing number of reports of RT ferromagnetism in undoped oxide thin films have been published, with nominal nonmagnetic electronic shell configurations (so-called “d^0^ magnetism”^7^). Various works suggested a charge transfer^8^ or donor-band ferromagnetism, both related to a Stoner splitting of the valence band. However, this has not been detected with XMCD^3^,^4^,^5^,^9^.

In order to provide magnetic moments, deviations from the empty or filled shell electron configuration must be present. This is consistent with experimental studies, correlating magnetism with oxygen vacancies and/or defects^10^,^11^,^12^,^13^. By analyzing a large number of experimental publications we found earlier, that the grain size, in particular the grain area to volume fraction, i.e. the specific grain boundary area s_GB_, plays an important role for ferromagnetism in doped and undoped ZnO nanostructures^14^,^15^.

In order to locate the origin for FM in our ZnO samples, we applied the local probe method of low energy muon spin relaxation (LE–μSR) here. Low energy positive muons are implanted into the host material and come to rest at interstitial lattice site due to their positive charge. Here they act as highly sensitive probes of magnetic fields originating from magnetic moments in their close proximity. More details on muon interaction with matter and the μSR method can be found in^16^,^17^.

## Experimental

Based on our experience of reproducing FM doped and undoped ZnO samples^18^,^19^,^20^,^21^,^22^,^23^,^24^,^25^,^26^,^27^ we produced two different ZnO thin film samples by means of the liquid ceramics method^19^. Zinc (II)-Butanoate with a concentration of 4 kg/m^3^ was used as a precursor. The precursor was deposited on 50 mm diameter sapphire disks with (102) orientation, polished on both sides, pre-cleaned by acetone and ethanol and pre-annealed to remove any remains of the cleaning solvent. Subsequently, the samples were annealed in a furnace in ambient conditions to 700 K (for 1 hour followed by a slow cool down, yielding the fine grained sample) and 1100 K (for 24 h, yielding the coarse grained sample). Two 10 × 10 × 1 mm^3^ and 5 × 5 × 1 mm^3^ commercial ZnO single crystals (Mateck Company, Germany) were used as nearly grain boundary free, nonmagnetic reference system for μSR and SQUID measurements.

## Results

**Figure 1:**
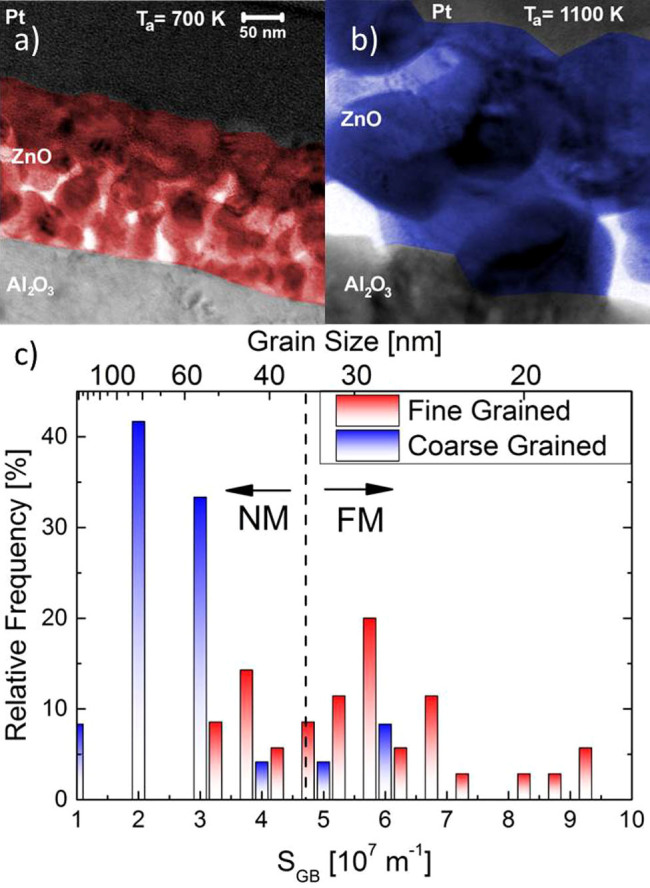
TEM micrographs of the fine (a) and the coarse grained (b) ZnO sample. (c) TEM based grain size and specific grain boundary area distribution. The dashed line marks the s_GB_ threshold limit for FM in undoped ZnO given in Ref. 14, separating approximately nonmagnetic (NM) and ferromagnetic (FM) regions. For the coarse grained sample (blue pillars), the majority of grains (85%) have a s_GB_ below the threshold limit. However, a small fraction of grains (15%) fulfills the FM condition. For the fine grained sample, the majority of grains (65%) have a s_GB_ above the threshold suggesting a higher FM response.

Annealing to 700 K resulted in a ZnO thin film with fine graining (small grains, labeled as FG in the following), shown in figure 1 a). Annealing to 1100 K resulted in a ZnO film with coarse graining (large grains, labeled as CG in the following), shown in figure 1 b). The ZnO films are trapped between the sapphire substrate (bottom part) and a protective Pt capping layer (top part), necessary for focused ion beam (FIB) TEM lamella preparation. In order to avoid substrate background influence on the μSR measurements, relatively large film thicknesses of 470 nm (CG) and 215 nm (FG) have been chosen.

A detailed analysis of the grain size distribution is shown in figure 1c), for the FG (red pillars) and the CG (blue pillars) thin films. The quantitative grain size distribution was determined by analyzing grains from multiple TEM micrographs. The specific grain boundary area s_GB_ was calculated as defined in Ref. 14: s_GB_ = 1.65·*a/D*, where *a* and *D* denote the grain aspect ratio and the grain diameter respectively. The s_GB_ distribution is given in figure 1 c) together with the respective grain sizes as a second scale. The average value of the s_GB_ is 5.32·10^7^ m^−1^ for the FG and 2.65·10^7^ m^−1^ for the CG sample, corresponding to an average grain size of 31 nm and 65 nm respectively. According to Straumal et. al.^14^ the empirical threshold s_GB _value for the occurrence of FM in nanograined ZnO systems is located in the vicinity of 5.0·10^−7^ m^−1^ and is marked as black dashed vertical line in figure 1 c). For the CG sample the grain size is narrowly distributed around its average value. However, a small grain fraction of about 10-15% is “critical”, exhibiting a s_GB_ value close to or above the threshold limit. This will supposedly lead to a small but sizeable sample magnetization. For the FG sample the s_GB_ distribution starts below the threshold limit, but stretches beyond it. The fraction of critical grains is 60%, suggesting a sample with more pronounced magnetization compared to the CG sample.

**Figure 2:**
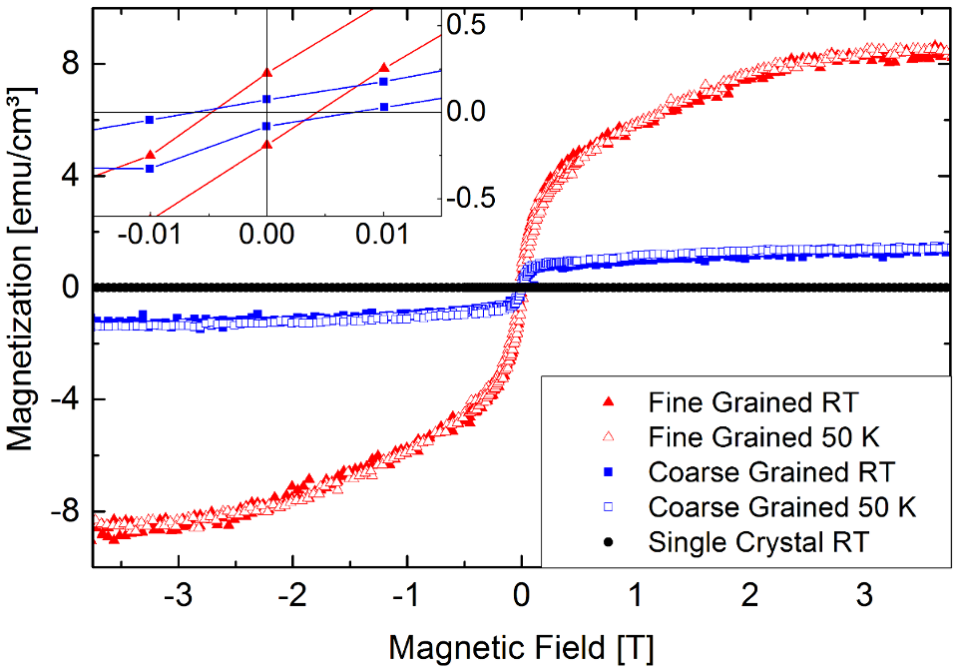
Temperature dependent SQUID magnetization curves for the fine grained (red triangles), and the coarse grained ZnO samples (blue squares). Even at RT, ferromagnetic magnetization curves with small but sizeable remanence and coercivitiy have been measured (see inset). For both sample types, SQUID loops measured at RT and 50 K show no significant difference which is an important feature identifying ferromagnetism in magnetic oxides. The supposedly nonmagnetic ZnO single crystal reference shows no significant magnetic features (black circles).

These expectations are confirmed by the corresponding SQUID measurements presented in figure 2. All magnetization curves have been corrected for the substrate diamagnetism. In order to avoid thickness dependent effects, the magnetization curves of the thin film samples have been normalized to the corresponding thin film volume, determined from the TEM based films thicknesses and the respective sample area. The magnetization curve of the single crystal has been normalized to the single crystal volume. The magnetization curves of the FG and CG samples have been measured at RT and at 50 K in order to confirm FM and to distinguish the hysteresis loops from temperature dependent (super–) paramagnetic curves^28^. As expected from the large fraction of “critical” grains, the FG sample shows the highest saturation magnetization with 8.3·emu/cm^3^, followed by the CG sample with 1.25 emu/cm^3^. The single crystal shows only a very small saturation magnetization of 2·10^−4^ emu/cm^3^. The magnetization curves at RT and 50 K are quite similar, which is an important indicator for true FM and a high Curie temperature T_C_. Furthermore, both magnetic samples exhibit a small but sizeable remanence and coercivity at RT, as shown in the inset of figure 2.

**Figure 3:**
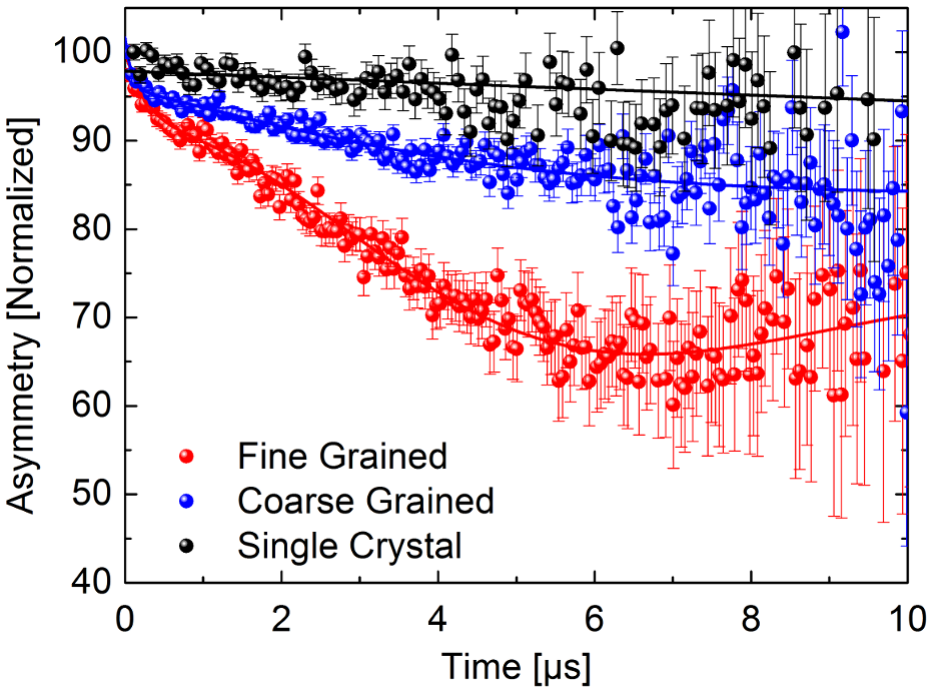
Averaged zero field μSR asymmetry, normalized to the initial detector asymmetry, for the single crystal (black dots), the coarse grained (blue dots), and the fine grained (red dots) ZnO sample. Plotted is the normalized detector asymmetry; the relaxing amplitude of the asymmetry is a measure for the magnetic volume fraction. The strongest relaxation is found for the fine grained sample (red dots), corresponding to a total magnetic volume fraction of about 35%. For the coarse grained sample (blue dots), the magnetic volume fraction is approx. 15%. The non–magnetic ZnO single crystal reference (black dots) shows no significant magnetic volume fraction.

Zero field muon spin relaxation (ZF–μSR) shows the depolarization of muons and the corresponding decay in the muon asymmetry spectrum^29^ due to the presence of an internal magnetic field distribution. Figure 3 shows the time dependent μSR asymmetry for the three samples. The corresponding decay functions were fitted using the program Musrfit^30^. While the single crystal shows no significant magnetic depolarization in the μSR signal, the thin film samples exhibit a clear damping. In addition, on a short timescale < 1 μs a fast damping of the signal is observed, related to a distribution of strong local magnetic fields, while for longer times the depolarization rate is reduced, related to an additional distribution of small magnetic fields.

For the FG sample, a superposition of a fast exponential decay, a Gaussian-Kubo-Toyabe (GKT) decay function^31^,^32^, and a constant term provides the best fit results:
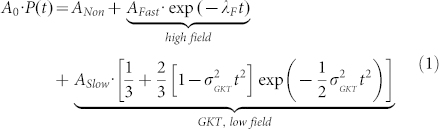
The exponential decay function corresponds to the quick depolarization of the muons related to high magnetic fields. It dominates the μSR asymmetry on the small time scale < 1 μs. The GKT decay function corresponds to muons residing in sample regions with low magnetic fields generated by densely packed, Gaussian distributed small magnetic moments.

From the fitting parameters A_i_ one can determine the respective volume fraction:


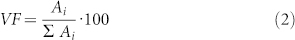
The corresponding volume fractions are VF_High_ = 9% for the high local magnetic field part, VF_Low_ = 26.5% for the low local magnetic field part, and VF_Non_ = 64.5% of the sample is nonmagnetic.

The asymmetry data of the CG sample could be best fitted by a combination of a fast exponential decay, a Voigtian–Kubo–Toyabe (VKT) decay function^33^, and a constant term related to the nonmagnetic sample volume fraction:


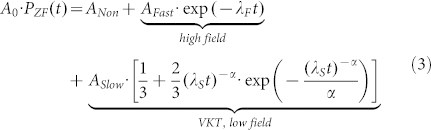
Similar to the results found for the FG sample, the exponential decay function corresponds to sample regions containing high magnetic fields. The VKT function is a generalized form of the GKT term in equation (1), considering a more dilute distribution of small magnetic moments within the sample volume. For α = 1 the VKT function corresponds to a Lorentzian magnetic moment distribution, for α = 2 one obtains the dense moment distribution case, the GKT function as presented in equation (1). Thus the VKT term is related to low magnetic fields generated by magnetic moments with a more dilute distribution. It should be noted here that although equations (1,3) provide information about the magnetic moment distribution, one cannot deduce whether these moments are of spin and/or orbital origin.

The corresponding volume fraction for the high local magnetic field part is VF_High_ = 5.8% VF_Low_ = 13.4% for the low local magnetic field part, while VF_Non_ = 80.8% of the CG sample is nonmagnetic.

From the respective decay parameters λ_F,S_ and σ the widths of the local magnetic field distribution, centered at zero field, can be determined using the gyromagnetic ratio of the muon^19^. The local magnetic fields are generated by magnetic moments located within the respective areas of the sample.

For the CG sample, the high field distribution width is 9.3 ± 0.2 mT, the low field distribution width is 0.14 ± 0.04 mT. For the FG sample, the high field distribution width is 8.7 ± 1.4 mT, the low field distribution width is 0.18 ± 0.02 mT.

The high magnetic field values are ~ 200 times smaller than the magnetization in bulk Fe (bcc) which is 2.2 T^34^, corresponding to ~ 2 μ_B_/Fe atom^34^. In a rough estimate the magnetization per magnetic moment carrying entity in ZnO, e.g. a vacancy site in grain boundaries, is in the order of ~ 0.01 μ_B_. Indeed the saturation magnetization of the CG sample is 200 times smaller than in bulk Fe (8.3 emu/cm^3^ compared to 1710 emu/cm^3^ see Ref. 35). The saturation magnetization of the FG sample is 1000 times smaller, which is related to the dilute distribution of magnetic moments as identified by the VKT term in equation (3).

No obvious dependence on the implantation depth of the muons inside the sample and the temperature could be detected (see supplementary figures SF1 and SF2); which is in agreement with the corresponding, temperature independent SQUID measurements. The distribution widths of internal magnetic fields within the magnetic volumes are similar for the CG and the FG sample, indicating equal internal magnetic moments. On the other hand, the (integrating) SQUID measurements show a reasonable difference in saturation magnetization. This is a direct consequence of the different μSR related magnetic volume fractions.

A possible complication in interpreting μSR spectra is the formation of so–called muonium, where the (positively charged) muon captures an electron from the ZnO host matrix: Mu = [μ^+^e^−^]. In ZnO, the muonium state can be identified by the appearance of additional precession frequencies in the μSR spectra measured in an applied magnetic field^36^ at low temperature (T<50K). At higher temperatures this state does not exist due to thermal ionization^37^. However, we did not observe such a muonium state at T = 50K, and can thus be ruled out as a cause for the magnetic signature we found in the ZF–μSR spectra of our samples.

## Discussion

**Figure 4:**
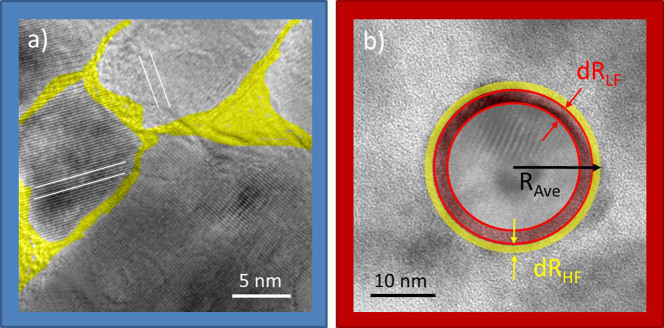
Selected TEM picture of a nanograin in the coarse (a) and the fine grained (b) sample. Crystallographic lattice planes are indicated as white lines. The suggested magnetic grain boundary region is marked yellow (dR_HF_), while the expected dipolar coupled proximity related magnetic region is marked red (dR_LF_), encasing the nonmagnetic inner grain region.

We developed a semi-quantitative microscopic model to simultaneously explain the μSR and SQUID measurements. As highlighted in Fig. 4a), the mainly amorphous grain boundary region^15^ has a range of approximately 1–2 nm in thickness (yellow). If one assumes that this GB region represents the μSR related high field magnetic volume fraction, then there is an intrinsic separation between this high field volume and neighboring low field regions were muons sense the declining stray fields.

In a spherical approximation we are able to estimate the effective thickness of the magnetic GB region and the adjacent low field region. We assume the actual ZnO grain as a nonmagnetic sphere with radius R_Ave_, which corresponds to the respective average grain size of the CG and FG sample. The high field region is located directly in the GB (yellowish colored sphere shell in figure 4 b). The low field region (reddish colored sphere shell in figure 4 b) is an “intermediate” region where muons sense stray fields from the actual magnetic grain boundary. The effective thickness for either spherical shell is named dR. Using dR, one can calculate the corresponding volume fraction for a given shell thickness. With the magnetic shell volume, V_m _ = 4π·R_ave_^2^·dR, one obtains the magnetic volume fraction V_m_/V_grain _ = (4π·R_ave_^2^·dR)/(4/3·π·R_ave_^3^) = 3·dR/R_ave_. Equating V_m_/V_grain_ with the respective μSR related VF and solving for dR yields the effective thickness of the high and low field sphere. The respective shell thickness of the high (dR_HF_) and low (dR_LF_) field regions are listed in table 1.

The estimated thickness of the regions containing high magnetic fields equals 0.95 nm (FG) and 1.3 nm (CG) respectively. The respective ranges are exactly in the range of the typical GB thicknesses of 1–2 nm. In addition, the estimated low field shell thickness is 3.1 nm (FG) and 2.8 nm (CG) respectively, which is a typical value for dipolar like fields decaying below the detection limit^29^.

This quantitative model based on the ZF–μSR results provides consistent results between the magnetic volume fractions of the FG and the CG sample and the respective fraction of “critical” grains estimated from TEM pictures.

**Figure 5:**
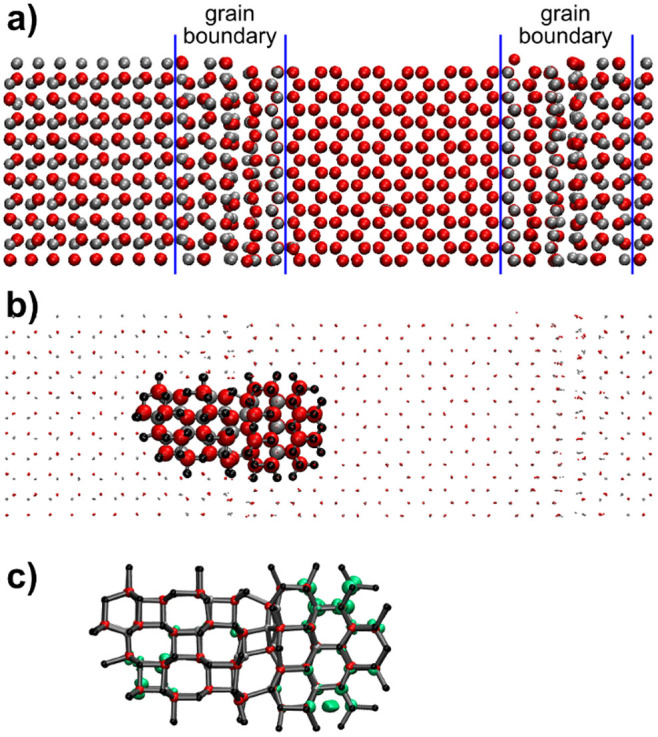
Electronic structure near a grain boundary in ZnO. a) atomistic model for the grain boundaries generated by molecular dynamics simulations (red: oxygen, grey: zinc); b) cluster of atoms for which the electronic structure was determined, embedded into an effective electrostatic field of point charges of the rest of the sample, dots symbolize point charges, balls the quantum cluster, black balls are zinc ions at the border of the quantum cluster treated by an all electron pseudo potential; c) spin density (light green) of the triplet state of the cluster.

To test the hypothesis that FM in ZnO indeed stems from magnetic moments located in the grain boundaries as claimed above, molecular dynamics simulations were performed to generate a realistic morphology for the disordered region near a GB. This GB region has been subsequently investigated using electronic structure theory (DFT), whether the existence of unpaired electrons in the grain boundaries is plausible. Figure 5a) shows an atomistic model of two grain boundaries between crystalline regions rotated by 90° with respect to each other, where the atomistic configuration at the GB has been generated in molecular dynamics. Then the electronic structure of a cluster with about 200 atoms has been computed using density functional theory in an effective electrostatic field formed by the rest of the simulated system. We find that the energy difference between the highest occupied molecular orbital (HOMO) and the lowest unoccupied molecular orbital (LUMO) amounts to more than 4 eV in calculations on bulk ZnO, which is reduced to zero for the sample containing the nearly disordered section at the grain boundary. Furthermore, the energy of the lowest magnetic triplet state of the cluster model for the grain boundary is only 0.2 eV higher than the closed shell ground state. These results suggest that the formation of unpaired electrons is possible at grain-boundaries and that geometries may exist, where such electrons are coupled ferromagnetically. This could be observed by the spin polarization shown in figure 5c).

In summary, we quantitatively estimated three different volume fractions in nanograined ZnO samples with a high field, low field and a non–magnetic fraction. The magnetic volume fractions increase with reduced grain size of the samples. On the other hand, the internal magnetic field distribution is independent of the grain size distribution. We present a magnetic shell model based on “magnetic” grain boundaries, as supported by DFT related theoretical calculations. This microscopic model is in very good agreement with the μSR results and also explains the total magnetizations of the samples. Our results open the way to a new view on the field of ferromagnetic semiconductors and could give rise to a new push into development of semiconductor based spintronic devices even without a TM doping intermediate step.

## Methods

To resolve the microscopic properties of the ZnO thin films, TEM measurements were performed on a Jeol JEM-4000FX microscope at acceleration voltage of 400 kV. In the same step energy dispersive x-ray spectroscopy (EDX) was used to exclude the presence of Fe, Ni, or Co contaminations in the samples. Their concentration was below 0.1 at.%.

Magnetic properties were measured on a MPMS-XL superconducting quantum interference device (SQUID) from Quantum Design. Magnetic fields were applied parallel to the sample surface.

Low energy muon spin relaxation (LE-μSR) was performed at the μE4 Low Energy Muon (LEM) beamline at the Swiss Muon Source (SμS), Paul Scherrer Institute, Switzerland^38^,^39^. Positive muons were implanted with initial 100% spin polarization parallel to the sample surface. The measurements were performed at different sample penetration depths (10 nm to 75 nm) and different temperatures (50 K, 100 K, and 250 K), and in zero field. Because we found no temperature or implantation depth dependence, μSR spectra presented in Fig. 3 were obtained by averaging in order to improve the signal to noise ratio.

To simulate the atomic disorder in the grain boundary region, we have performed molecular dynamics simulations of 4800 atoms in periodic box with initial size 2.6 × 2.6 × 8.4 nm^3^ and two grain boundaries. The interactions between the atoms are calculated by the Buckingham and Coulomb potentials with the parameter set from^40^,^41^. The simulation was done at constant pressure p = 1 bar under NPT conditions. The system was first equilibrated at 300 K for 0.5 ns, then heated to 2700 K and equilibrated for 1 ns. Afterward the system was cooled to 300 K and equilibrated for 1 ns. During the simulation, premelting at the grain boundaries is observed.

The quantum chemical calculations have been performed with the program package Turbomole (V6.4 2012, a development of University of Karlsruhe and Forschungszentrum Karlsruhe GmbH, 1989–2007. *Turbomole GmbH, since 2007; available from* http://turbomole.com). An embedded cluster approach was used, i.e. a Zn_51_O_92_Zn(PP)_110_ quantum cluster was embedded in an extended point charge field (see figure 5 b). The point charge field was generated from the simulation cell of the MD calculations. Dipole and quadrupole moments were removed by an Evjen type procedure and one neighbor cell added in each direction. Density functional theory (BP86/def2-SVP) has been used throughout. In all calculations, the RI-J approximation was used for the interelectronic Coulomb term. The Zn(PP) ions form the border to the point charge field and are equipped by large core pseudopotentials^42^.

## Author Contributions

T.T., P.A., E.G., T.P., H.L., Z.S., and A.S. performed μSR experiments and corresponding data analysis. T.T. and Y.-C.C. performed SQUID measurements and corresponding data analysis. A.M. and the STEM Group at the MPI-IS Stuttgart, Germany have performed the TEM measurements. P.B.S., B.B.S, and S.G.P. prepared ZnO thin film samples. K.F., W.W., and D.D. performed electronic structure, electron and molecular dynamics calculations. T.T. and E.G. wrote the manuscript and physical modeling with contributions from the above mentioned experts. B.B. and G.S. assisted further discussions and final proof reading.

## Supplementary Material

Supplementary Information Supporting Information

## Figures and Tables

**Table 1:** Magnetic volume fractions and the related shell thicknesses for each ferromagnetic sample

	FG (R_ave_ = 31 nm)	CG (R_ave_ = 65 nm)
dR_HF_	For V_m_/V_grain_ = 9%; 0.95 nm	For V_m_/V_grain_ = 5.7%; 1.3 nm
dR_LF_	For V_m_/V_grain_ = 26.5%; 3.1 nm	For V_m_/V_grain_ = 13.4%; 2.8 nm
