# Supplementary material for: Interfacial dominated ferromagnetism in nanograined ZnO: a μSR and DFT study
Source: Sci Rep. 2015 Mar 9;5:8871. doi: 10.1038/srep08871 (PMC4352909; doi:10.1038/srep08871)
Supplement: Supplementary Information — Supporting Information [file srep08871-s1.pdf]

Supplementary Information for:

Interfacial dominated ferromagnetism in nanograined ZnO: a μSR and DFT study

Thomas Tietze[1], Patrick Audehm[1], Yu-Chun Chen[1], Gisela Schütz[1,*], Boris B. Straumal[2,3,6], Svetlana G. Protasova[3], Andrey A. Mazilkin[3], Petr B. Straumal[4], Thomas Prokscha[5], Hubertus Luetkens[5], Zaher Salman[5], Andreas Suter[5], Brigitte Baretzky[6], Karin Fink[6], Wolfgang Wenzel[6], Denis Danilov[6], Eberhard Goering[1]

* Email: schuetz@is.mpg.de

[1]Max-Planck-Institute for Intelligent Systems, Heisenbergstr. 3, D-70569 Stuttgart, Germany

[2]Moscow Institute of Physics and Technology (State University), Institutskii per. 9, 141700 Dolgoprudny, Russia

[3]Institute of Solid State Physics, Russian Academy of Sciences, Ac. Ossipyan str. 2, 142432 Chernogolovka, Russia

[4]National Research Technological University "MISiS", Leninsky prosp. 4, 119991 Moscow, Russia

[5]Laboratory for Muon Spin Spectroscopy, Paul Scherrer Institut, CH-5232 Villigen, Switzerland

[6]Karlsruhe Institute of Technology, Institute of Nanotechnology, Hermann-von-Helmholtz-Platz 1, D-76344 Eggenstein-Leopoldshafen, Germany

We performed ZF-μSR measurements for a set of temperatures, 50 K, 100 K, and 250 K, and varying the implantation depth in order to probe possible thickness related effects. The high magnetic field distribution width, stemming from the exponential decay function in equations (1) and (2) were determined using a Fourier transform and estimating the half width at half maximum (HWHM) of the corresponding Lorentz distribution. The low magnetic field distribution width was determined from the fitparameters of the VKT/GKT decay functions in (1)/(2) respectively. The temperature and implantation depth dependent magnetic field distribution widths are shown in SF1 for the fine and in SF2 for the coarse grained sample. For both samples, we did not observe an obvious dependence of temperature or implantation depth. Thus the respective spectra were averaged to obtain the ZF-μSR presented in figure 3.

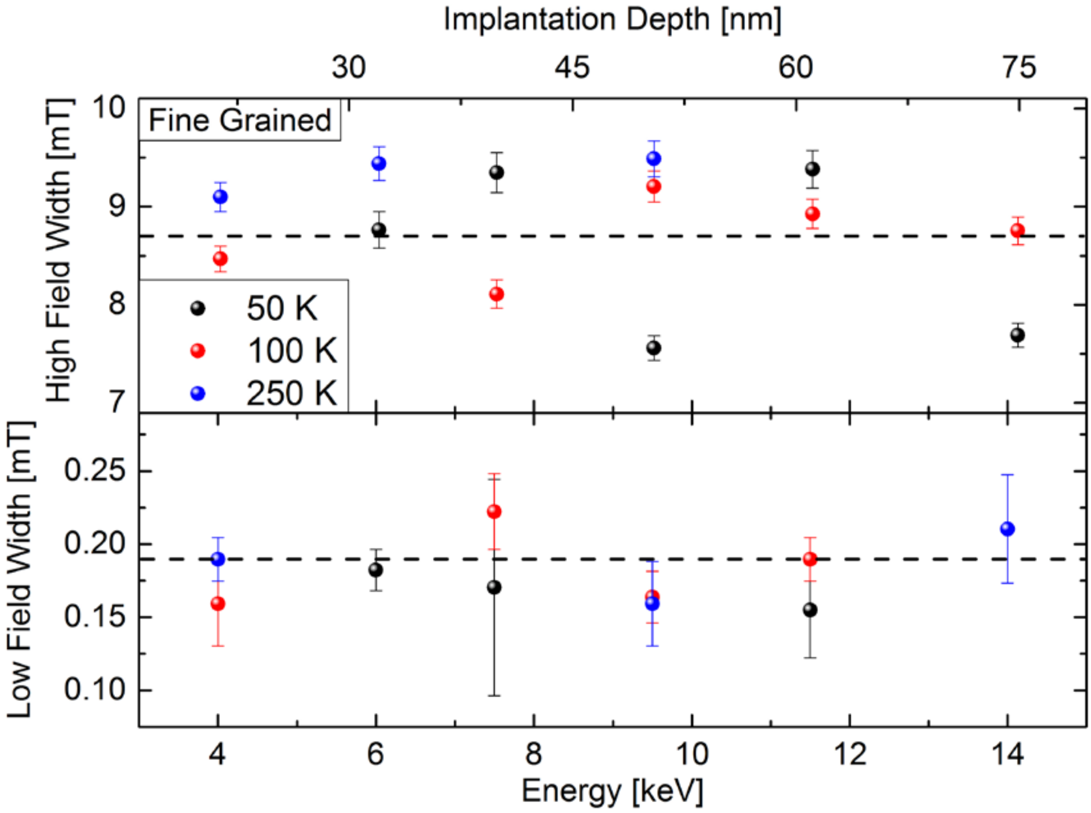

SF 1: The depth and temperature dependence of the magnetic field distribution width for the fine grained sample shows no obvious trend line, neither for the high (top) nor for the low field (bottom) distribution width. The average field distribution width is plotted as dashed line respectively.

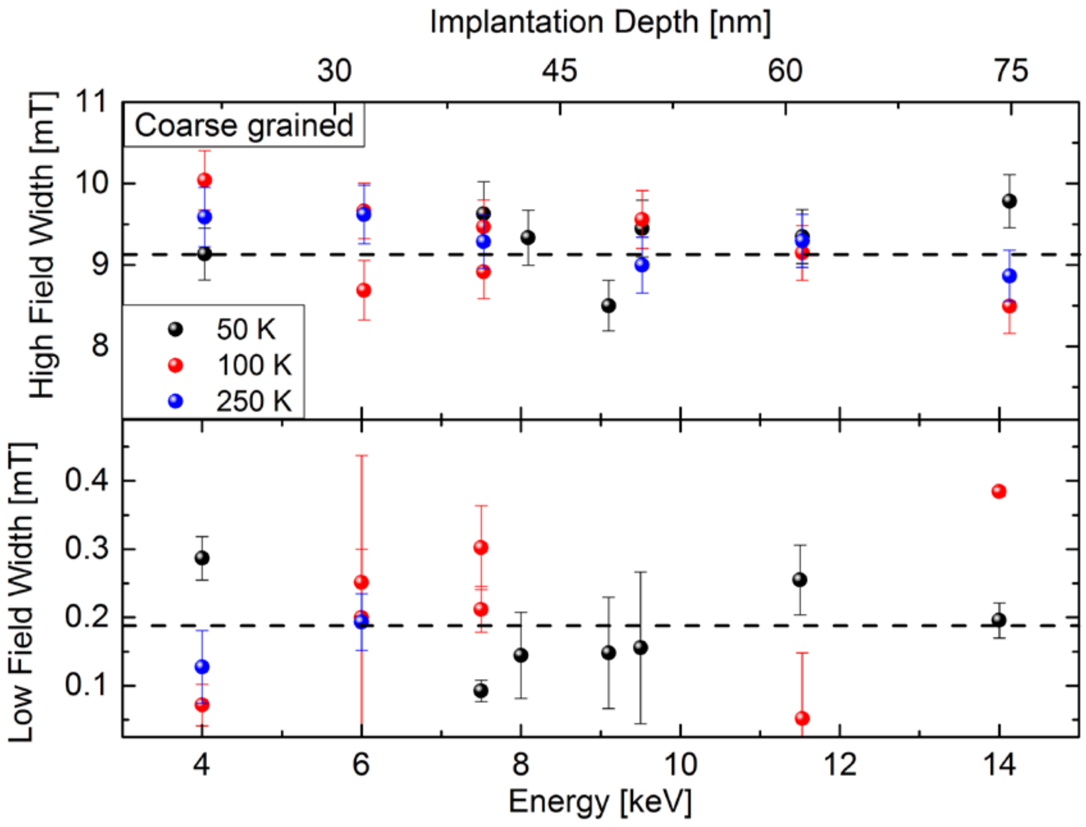

SF 2: For the coarse grained sample there is no significant implantation depth or temperature for the high (top) and low field distribution width. The field distribution width is constant within a certain value range. The respective average field distribution widths were plotted as dashed lines.
